# Chemical Composition, Acetylcholinesterase-Inhibitory Potential and Antioxidant Activity of Essential Oils from Three Populations of *Parthenium hysterophorus* L. in Ecuador

**DOI:** 10.3390/molecules30132712

**Published:** 2025-06-24

**Authors:** James Calva, María Belén Cuenca, Andrea León, Ángel Benítez

**Affiliations:** 1 Departamento de Química, Universidad Técnica Particular de Loja, Loja 1101608, Ecuador; 2 Carrera de Bioquímica y Farmacia, Universidad Técnica Particular de Loja, Loja 1101608, Ecuador; mbcuenca2@utpl.edu.ec (M.B.C.); agleon11@utpl.edu.ec (A.L.); 3 Biodiversidad de Ecosistemas Tropicales-BIETROP, Herbario HUTPL, Departamento de Ciencias Biológicas y Agropecuarias, Universidad Técnica Particular de Loja (UTPL), Loja 1101608, Ecuador; arbenitez@utpl.edu.ec

**Keywords:** *Parthenium hysteroporus*, chemical composition, AChE, gas chromatography

## Abstract

In this study, we analyzed the essential oils (EOs) extracted by steam distillation from the leaves and flowers of *Parthenium hysterophorus* L., grown in three different locations in southern Ecuador: Espíndola (ESP), Loja (LOJ) and Quilanga (QUI). Approximately 97.45%, 98.27% and 95.99% of the oil constituents were identified using gas chromatography–mass spectrometry (GC-MS) and gas chromatography–flame ionization detection (GC-FID). Sesquiterpene hydrocarbons were predominant in the EOs. The most representative constituents of the sample from ESP were germacrene D (35.08%), myrcene (11.32%), (*E*)-β-ocimene (10.21%), (*E*)-caryophyllene (7.90%), germacra-4(15),5,10(14)-trien-1-α-ol (5.18%) and (*E*, *E*)-α-farnesene (4.99%), with an AChE IC_50_ of 14.78 and with 16.38 and 93.16 µg/mL from ABTS and DPPH, respectively. The EOs from LOJ were characterized by the abundant presence of germacrene D (28.30%), myrcene (13.95%), (E)-β-ocimene (10.51%) and isolongifolan-7-α-ol (8.26%), with an AChE IC_50_ of 16.65 and with 36.18 and 158.43 µg/mL from ABTS and DPPH, respectively. Finally, the EOs from QUI contained germacrene D (32.20%), myrcene (13.36%), (E)-β-ocimene (8.54%), (*E*, *E*)-α-farnesene (6.77%), germacra-4(15),5,10(14)-trien-1-α-ol (5.69%) and (E)-caryophyllene (5.37%), with an AChE IC_50_ of 10.69 and with 14.50 and 92.98 µg/mL from ABTS and DPPH, respectively. This study identifies chemotypic variation in *P. hysterophorus* collected from different locations and, for the first time, the AChE inhibitor was evaluated. These findings provide a scientific basis for the clinical application of *P. hysterophorus* EOs in the future treatment of Alzheimer’s disease.

## 1. Introduction

Medicinal plants have long played a fundamental role in human development and the interaction with the surrounding environment [1]. Throughout history, these plants have been utilized in various aspects such as food, clothing and construction and, notably, for therapeutic purposes to combat illnesses and promote physical well-being [2]. In 2020, more than 50,000 species of plants with therapeutic effects were reported, accounting for approximately 10% of all known vascular plant species worldwide. Moreover, the utilization of these species is advancing due to the development of new technologies and ongoing research into their properties. This effort aims to identify and characterize the active compounds within medicinal plants and to determine their specific influence in various fields, particularly pharmacology and medicine [3].

The Asteraceae family comprises a large number of plant species used for medicinal, agricultural and industrial purposes. It is characterized by its diverse array of large flowers and includes approximately 23,000 species distributed among over 1620 genera. These plants are found worldwide, particularly in Arctic, tropical and desert regions [4]. In Ecuador, 310 endemic groups of Asteraceae in the flora have been reported. Furthermore, the family is taxonomically diverse, and it is divided into four subfamilies (Asteroideae, Barnadesioideae, Cichorioideae and Mutisioideae), 16 tribes and 89 genera [5].

Parthenium is a genus of phanerogamous plants within the family Asteraceae that comprises 249 species and is considered a tropical American plant [6]. The species *P. hysterophorus* L., commonly known as “bitter broom” or “escoba amarga” [7], is a branched herbaceous plant with 60-80 cm tall and ash-green herbs. It grows abundantly in many places, including public lawns, forests, flood plains, agriculture, urban areas, overgrazed pastures, roadsides, railway tracks and residential plots. Although it can grow in most soil types, this weed is most commonly found in clay loam and alkaline soils [8]. In Ecuador, it is a native herb and is found at altitudes between 500 and 1500 m in the coastal and sierra regions [9]. It is characterized by rapid growth and rapid expansion, its leaves are lobed, and it has small creamy white flowers grouped in clusters [10]. Studies have shown that this species has allergenic potential and can be considered harmful to health as it can cause allergic reactions, dermatitis and respiratory issues in some individuals [11].

The chemical composition of EO varies considerably depending on factors such as geographical location, climate, soil type and extraction methods [12]. Previous studies on *P. hysterophorus* have revealed a predominant sesquiterpene hydrocarbon composition, with germacrene, trans-β-ocimene, β-myrcene, germacrene D and geraniol as the major bioactive components. It has medicinal properties such as anti-inflammatory, antimicrobial and antioxidant effects, among others [13,14]. However, there are discrepancies in terms of the dominant compounds at different sites, highlighting the need for analysis in different sectors. Therefore, while some studies emphasize sesquiterpenes as the primary components [15], others highlight monoterpenes as key contributors to the oil’s bioactivity [16]. Such variations necessitate further investigation into how environmental conditions influence the chemical makeup of *P. hysterophorus* essential oils.

Alzheimer’s disease is a progressive neurodegenerative disorder characterized by cognitive decline and cholinergic dysfunction [17]. Therefore, the investigation of AChE inhibitors is crucial for its treatment, as these compounds prevent the degradation of acetylcholine and enhance cholinergic transmission [18]. Recent studies have demonstrated that certain terpenoid-rich EOs exhibit potent AChE-inhibitory activity, making them promising candidates for drug development [19]. In our study, the results of the AChE assay showed moderate-to-high inhibitory activity. However, these findings are preliminary and further research is required to understand their mechanism of action.

The aim of this study was to characterize the chemical composition of EOs from *P. hysterophorus* at different collection sites in southern Ecuador and evaluate their AChE-inhibitory and antioxidant activities. We hypothesize that the chemical composition of essential oils (EOs) extracted from *P. hysterophorus* L. is influenced by geographical origin, and that this affects their ability to inhibit acetylcholinesterase (AChE) and demonstrate antioxidant activity. This research goes beyond expanding our scientific understanding of the bioactive potential of *P. hysterophorus*. It also aims to lay a strong foundation for its potential clinical application in the treatment of Alzheimer’s disease, opening new therapeutic perspectives and contributing to the development of innovative and effective treatments for this neurodegenerative disease.

## 2. Results

### 2.1. Obtaining the Essential Oil

The EOs of aerial parts of *P. hysterophorus* L. were obtained by steam distillation for 2 h. The oil showed a yellowish color in the three distillations and an oily phase at room temperature, after which it was stored in amber glass vials. The EO yields per collection sector were 0.013% for Espíndola, 0.015% for Loja and 0.017% for Quilanga.

### 2.2. Chemical Composition

**Figure 1:**
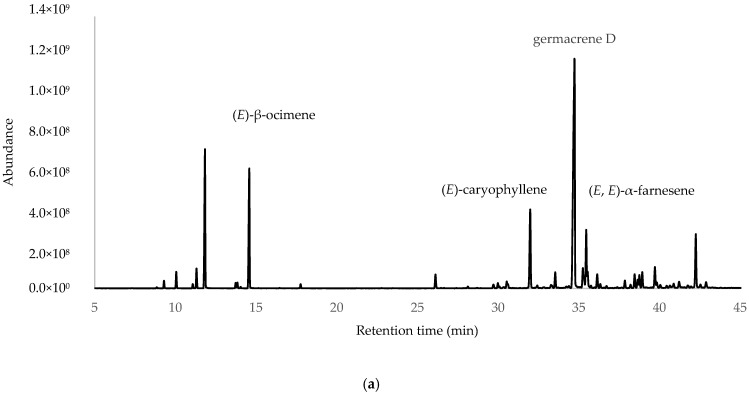

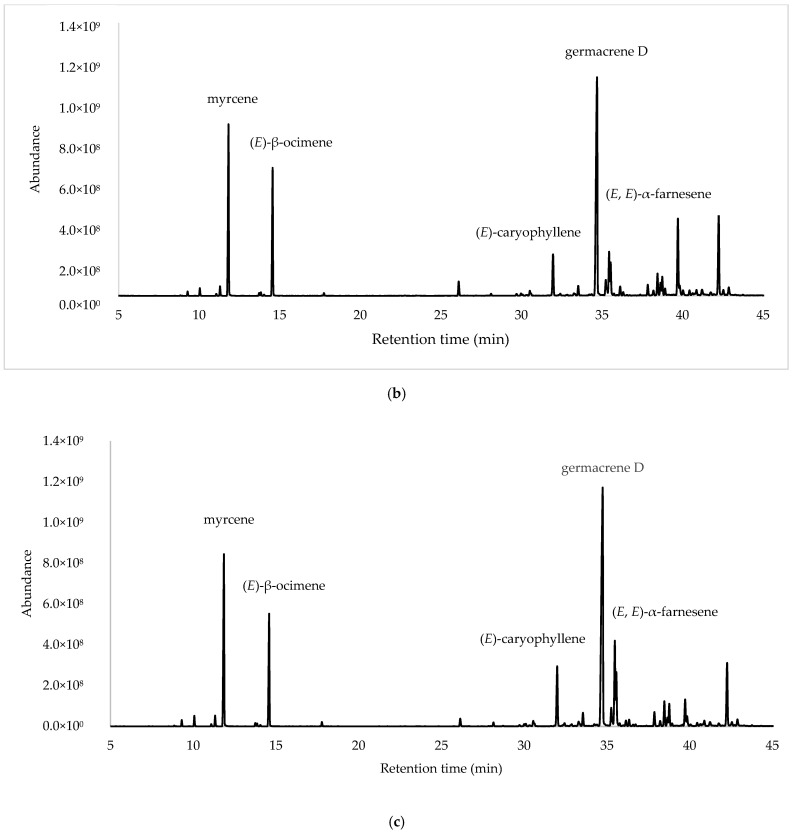
Gas chromatograms of *Parthenium hysterophorus* L. EOs from (**a**) Espíndola, (**b**) Loja and (**c**) Quilanga obtained using 5-phenyl-methylpolysiloxane column.

After the integration of the chromatograms, a total of 70 compounds were identified, corresponding to 97.45%, 98.27% and 95.99% of the total oil constituents on the DB-5ms column, belonging to the samples obtained from ESP, LOJ and QUI, respectively. The most abundant compounds were germacrene D (35.27%, 28.30% and 32.22%); myrcene (11.33%, 13.95% and 13.36%); (*E*)-β-ocimene (10.21%, 10.51% and 8.54%); (*E*)-caryophyllene (7.93%, 3.98% and 5.37%); and (*E*, *E*)-α-farnesene (4.99%, 3.64% and 6.77%) (Table 1, Figure 1 and Supplementary Materials Section). The essential oil was mainly composed of hydrocarbonated and oxygenated sesquiterpenes and hydrocarbonated monoterpenes.

### 2.3. Anticholinesterase Activity

**Figure 2:**
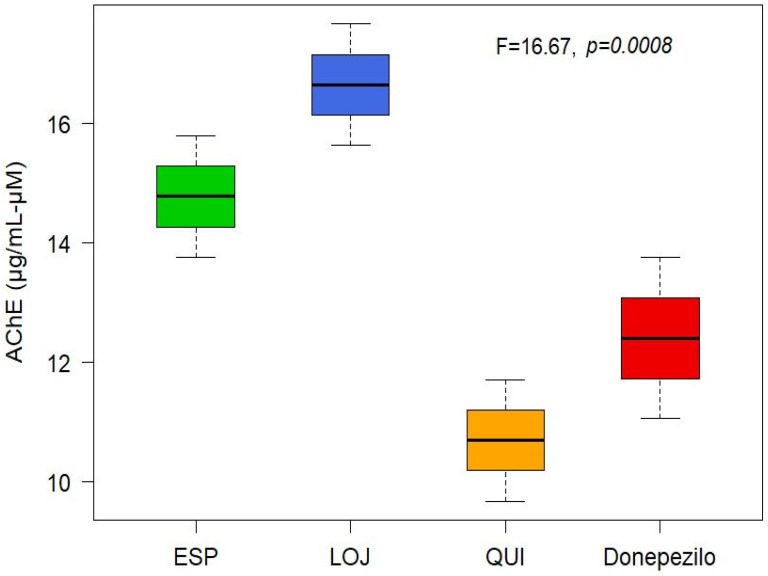
Boxplot of AChE-inhibitory activity of *Parthenium hysterophorus* L.

**Figure 3:**
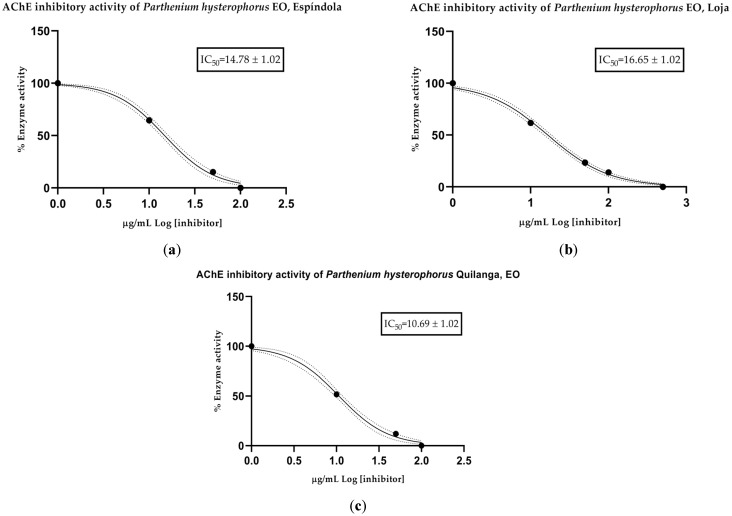
AChE-inhibitory activity of *Parthenium hysterophorus* L. essential oils from (**a**) Espíndola, (**b**) Loja and (**c**) Quilanga; solid lines = trend lines; dotted lines = 95% confidence intervals.

The anticholinesterase (anti-AChE) activity of the EOs was determined spectrophotometrically. Mean inhibitory concentration (IC_50_) values were calculated for each collection sector: 14.78 µg/mL for Espíndola, 16.65 µg/mL for Loja and 10.69 µg/mL for Quilanga. The positive control, donepezil, had an IC_50_ value of 12.40 µg/mL. According to the scale of values for anticholinesterase activity, the results indicate that the EOs have a moderate-to-high potency to inhibit the AChE enzyme (Figure 2 and Figure 3). This inhibitory capacity is relevant due to its potential application in the treatment of neurodegenerative diseases such as Alzheimer’s disease, as AChE inhibition favors the transmission of nerve signals by maintaining adequate levels of acetylcholine.

### 2.4. Antioxidant Activity

**Figure 4:**
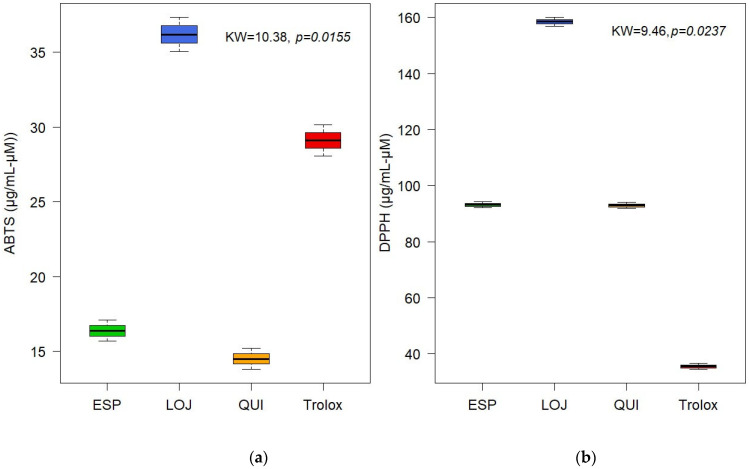
Boxplot of antioxidant activity of essential oils from *Parthenium hysterophorus* L. evaluated by (**a**) ABTS and (**b**) DPPH.

The antioxidant activity of the EOs was evaluated using the ABTS and DPPH methods. The results showed moderate activity, with IC_50_ values for Quilanga of 14.50 µg/mL and 92.98 µM, for Espíndola of 16.38 µg/mL and 93.16 µM and for Loja of 36.18 µg/mL and 158.43 µM, for ABTS and DPPH, respectively. These values were compared to the positive control Trolox, which showed a significant but lower antioxidant capacity compared to other essential oils previously studied. This finding suggests that *P. hysterophorus* L. (Figure 4) EOs could be useful in health and food industry applications, although their efficacy will depend on the dose and context of use.

Table 2 summarizes the mean inhibitory concentration values (IC_50_) obtained in biological assays performed on *P. hysterophorus* EOs collected in three different areas of southern Ecuador. These values represent the necessary concentration of each sample to inhibit 50% of the activity of the enzymes evaluated (AChE) or to neutralize 50% of the free radicals (DPPH and ABTS).

### 2.5. Multivariate Analysis

**Figure 5:**
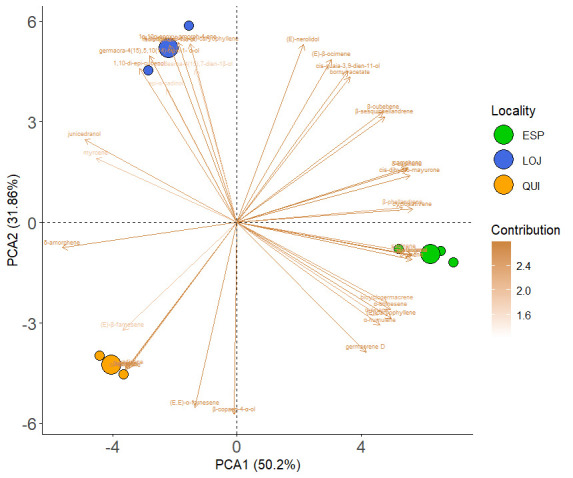
Results of the PCA for the DB5-ms of the main compounds of *Parthenium hysterophorus* L. in the different collection localities.

Statistical analysis using principal component analysis (PCA) revealed variations in the chemical composition of the essential oil between the three collection sectors. Through the use of the DB-5ms column, component 1 explained 68.13% of the variance, while component 2 explained 31.87%. Thus, a stronger clustering of compounds was observed within each locality, while greater variation was detected between localities. For example, in Loja, the major compounds were isolongifolan-7-α-ol and (*E*)-β-ocimene; in Espíndola, (*E*)-caryophyllene and germacrene D predominated; and in Quilanga, δ-amorphene and (*E*, *E*)-α-farnesene were the most abundant (Figure 5). These results were confirmed by the PERMANOVA analysis, which showed significant differences in chemical compounds among the three localities (F = 118.5, *p* = 0.0039).

## 3. Discussion

The essential oils obtained from the leaves of *P. hysterophorus* L. exhibit different chemical profiles and biological activities for the populations studied. Chemical analysis identified a total of 70 compounds, with germacrene D (31.86%) being the most abundant compound, followed by myrcene (12.88%), (*E*)-β-ocimene (9.75%), (*E*)-caryophyllene (5.75%) and (*E*, *E*)-α-farnesene (5.13%). These results were consistent with previous studies on the genus Parthenium, where sesquiterpenes and monoterpenes have been reported as the dominant components in EOs [21].

The high concentration of germacrene D, a sesquiterpene known for its antimicrobial, anti-inflammatory and insecticidal properties, was consistent with that reported by Miranda et al. (2014) [22], who identified this compound as a major constituent of *P. hysterophorus* EO, with percentages of germacrene D (35.9%), trans-ocimene (8.5%) and myrcene (7.6%). In our study, the percentage of germacrene D (31.86%) was significantly higher than the 8.4% reported by Chandra et al. (2016) [14]. This variation can be affected by environmental factors such as altitude, temperature and soil composition, which are known to affect the biosynthesis of secondary metabolites in plants [23]. Although quantitative differences exist, the general trend observed in previous studies is similar to ours, reinforcing the validity of our findings. Moreover, other species within the genus also show a similar chemical composition. For instance, *Parthenium argentatum* Gray is mainly composed of germacrene D, bicyclogermacrene, elemol, γ-eudesmol and β-eudesmol [24]. The presence of myrcene as one of the main compounds further enhances the pharmacological potential of *P. hysterophorus,* as according to recent research, it has antioxidant, anti-inflammatory, anxiolytic, anti-aging and analgesic properties [16,25].

The antioxidant activity of the EOs was evaluated by the DPPH and ABTS assays. The EOs exhibited moderate scavenging activity, with IC_50_ values indicating lower potency compared to antioxidants such as Trolox, used as the positive control. However, our results are consistent with those of Iqbal et al. (2022) [26], who reported similar antioxidant capacities for leaf extracts of the same species. The moderate antioxidant activity may be attributed to the presence of oxygenated sesquiterpenes and monoterpenes, which are known to neutralize free radicals via hydrogen donation or electron transfer mechanisms [27]. Nevertheless, the relatively low levels of these compounds in the EOs (17.24% monoterpenes and <4% oxygenated sesquiterpenes) probably limited their overall efficacy. These results are comparable to a study conducted on the methanolic extract *P. hysteroporus* in India, which reported moderate antioxidant activity, with ABTS (IC_50_ of 33.6 ± 10.6 µg/mL) and DPPH (IC_50_ of 60.2 ± 14.09 µg/mL) from a positive control of 54.4 ± 10.22 µg/mL [28]. Therefore, it can be inferred that the EOs demonstrate higher antioxidant activity compared to the extracts, which may be due to the differences in extraction efficiency and compound solubility in the respective assay systems [28]. Future studies should focus on optimizing extraction methods to enhance the yield and concentration of antioxidant-rich fractions for potential applications in the pharmaceutical industry.

In the present study, the anticholinesterase activity of *P. hysterophorus* L. EOs was reported for the first time, and the observed activity can be attributed to the presence of sesquiterpenes such as germacrene D and (*E*)-caryophyllene, which have been shown to interact with the active site of AChE [29]. Although the IC_50_ values obtained in this study are higher than those of synthetic inhibitors such as donepezil (12.40 µg/mL), they highlight the therapeutic potential of *P. hysterophorus* essential oil as a complementary treatment option. This is in agreement with previous research on other species of the same family, such as the study by Lan et al. (2022) [19] in which it was found that the EO obtained from the flowers of *Chrysanthenum parthenium* had a higher inhibitory capacity against the AChE enzyme, with an IC_50_ value of 5.51 μg/mL. This may be explained by the fact that *Chrysanthenum parthenium* contains compounds such as parthenolide which have been shown to have potent AChE-inhibitory activity [30]. Further studies are needed to isolate and characterize the specific compounds responsible for this activity.

The results for antioxidant activity and AChE inhibition showed a clear correlation with the geographical origin of the samples. EOs collected in warmer regions such as QUI and ESP exhibited higher antioxidant activity and AChE inhibition compared to the LOJ samples, with this site being located in a cooler temperate zone. Studies have shown that higher temperatures can increase the production of secondary metabolites such as alkaloids and essences. For instance, a study published in *New Phytologist* reported that plants grow taller under warmer conditions to access more light and maintain an adequate carbon balance. This physiological adaptation not only enhances photosynthetic efficiency but may also promote the synthesis of bioactive chemical compounds [31].

The PCA and PERMANOVA results reflect the variability in the composition of the EOs between the three localities. This variability may be explained by environmental factors such as altitude, soil type and climatic conditions, which influence the biosynthesis of secondary metabolites [32]. Previous studies have shown that changes in temperature, humidity and ultraviolet radiation can alter the volatile profile in aromatic plant species [33,34] supporting our findings.

This study provides a preliminary foundation based on the in vitro inhibition of AChE, a key biomarker in the treatment of Alzheimer’s disease, and the AChE-inhibitory activity and moderate antioxidant properties of the EOs open new possibilities for future research. It is recommended to isolate and evaluate bioactive compounds for pharmacological assays, to explore potential synergies with other natural products or synthetic drugs and to perform in vivo studies to assess safety, efficacy and mechanisms of action. Furthermore, given that this species is invasive and has allelopathic effects, it is crucial to consider its ecological impact on agricultural and natural ecosystems [12]. These findings contribute to the growing body of knowledge on the genus Parthenium and open new avenues for research into its therapeutic applications. By addressing the limitations identified in this study, future investigations can unlock the full potential of this versatile plant species as a source of bioactive compounds with pharmaceutical relevance.

## 4. Methodology

### 4.1. Plant Material and Collection

Fresh aerial parts of *Parthenium hysterophorus* L. were collected in three different sectors in the south of Ecuador. The first collection was in the parish of El Ingenio, Espíndola, at an altitude of 1260 m a.s.l. (coordinates 4°21′23′′ S and 79°25′48′′ W). The second collection was carried out in the sector Amable María, Loja, at an altitude of 2094 m a.s.l. (coordinates 3°57′24′′ S and 79°12′48′′ W). Finally, the third collection was in the community of Las Aradas, Quilanga, at an altitude of 1240 m a.s.l. (coordinates 4°21′55′′ S and 79°25′34′′ W). The plant material was identified by Jorge Armijos, botanist at the UTPL Herbarium. Voucher specimens were deposited under the following codes: 14994 (ESP), 14995 (LOJ) and 14996 (QUI). Collection was conducted under a research permit of the Ministry of Environment, Water and Ecological Transition of Ecuador, with the code MAATE-DBI-CM-2022-0248.

### 4.2. Essential Oil Extraction

Essential oils (EOs) were extracted using steam distillation in a modified Dean–Stark apparatus at 95 °C for 3 h. This method was selected for its simplicity and relatively low investment requirements [35]. Fresh plant materials (approximately 3173 g ESP, 3109 g LOJ and 3146 g QUI) were used. The EOs were dehydrated with anhydrous sodium sulfate (Sigma-Aldrich, St. Louis, MO, USA) and stored in a glass vial at −4 °C to prevent the loss and alteration of their chemical composition. This procedure was carried out in triplicate for each collection sector [36]. Subsequently, the EO yield was calculated according to the methodology described by León et al. (2015) [37].

### 4.3. Chemical Composition Analysis

#### 4.3.1. Qualitative Analysis

The qualitative analysis was performed using a Thermo Scientific Gas Chromatograph Trace 1310 coupled with an ISQ7000 (Thermo Fisher Scientific, Wal-than, MA, USA) single-quadrupole mass spectrometer (GC-MS). A DB-5ms capillary column (5% phenyl-methylpolysiloxane, 30 m × 0.25 mm inner diameter, 0.25 μm film thickness) was employed for separation. The oven temperature programming started at 60 °C for 5 min, followed by an increase of 2 °C/min to 100 °C, and then a gradient of 3 °C/min to 150 °C, followed by a gradient of 5 °C/min to 200 °C. Finally, the temperature was increased at 15 °C/min to 230 °C and held for 3 min. Ultra-pure helium was used as carrier gas at a flow rate of 1 mL/min. Samples were prepared by diluting 10 µL of essential oil in 990 µL of dichloromethane (1% *v*/*v*) and were analyzed under split injection mode (split ratio 40:1) [36].

Compounds were identified by comparing retention indices and mass spectra with data from Adams (2007) [20] and the NIST Mass Spectra Library [38]. The calculated linear retention index (LRI) was determined by comparison with data from the literature and determined following the method of Van Den Dool and Kratz [39] using a mixture of η-alkanes from C_9_ to C_25_ (Sigma-Aldrich, St. Louis, MO, USA) injected under the same chromatographic conditions as the EO samples.

#### 4.3.2. Quantitative Analysis

The quantitative analysis was performed using the same GC system that was used in the qualitative analysis, using a flame ionization detector (GC-FID). All the conditions—thermal, column and gas flow—were the same as in GC-MS analysis, and the quantification was performed using peak area normalization.

### 4.4. Anticholinesterase Activity

The AChE-inhibitory activity was determined spectrophotometrically using the Ellman colorimetric method. The reaction mixture contained 40 µL of Tris buffer, 20 µL of EO, 20 µL of acetylthiocholine and 100 µL of DTNB reagent. Donepezil hydrochloride was used as a positive control [40]. The samples were pre-incubated at 25 °C for 3 min with continuous shaking. Subsequently, 20 µL of AChE at a concentration of 0.5 U/mL was added to initiate the reaction. Absorbance was measured at 405 nm after 1 h using a microplate reader (BioTek Epoch 2). The half inhibitory concentration was derived from a non-linear regression model (normalized response vs. log inhibitor–variable slope). The IC_50_ values were calculated using GraphPad Prism v8.0.1. Finally, 10 mg of the *P. hysterophorus* L. EOs was dissolved in 1 mL of MeOH.

### 4.5. Antioxidant Activity

#### 4.5.1. DPPH Assay

The DPPH (2,2-diphenyl-1-picrylhydrazyl) radical scavenging assay was carried out following the methodology established by Taipong et al. (2006) [41]. The working solution was prepared by diluting 24 mg of DPPH in 100 mL of MeOH. The sample was then stabilized in a BioTek Epoch 2 microplate reader (BIOTEK, Winooski, VT, USA), until an absorbance of 1.1 ± 0.01 was obtained at a wavelength of 515 nm. Subsequently, DPPH and EOs were mixed at different concentrations, ranging from 1.05 to 0.25 mg/mL. The mixture was placed in a 96-well microplate, adding 270 µL of the working solution adjusted with DPPH and 30 µL of the EOs. The reaction was monitored at a length of 515 nm for 1 h at room temperature. Trolox was used as a positive control and MeOH as a blank. The results are expressed in terms of 50% radical scavenging concentration (IC_50_), and curve fitting was conducted with GraphPad Prism v-8.0.1 software.

#### 4.5.2. ABTS Assay

This assay was based on the protocol of Arnao [42], with some variations proposed by Thaipong et al. (2006) [41]. A stock solution was prepared by mixing equal volumes of ABTS radical (7.4 µm) and potassium persulphate (2.6 µm). The solution was stirred constantly for 12 h at room temperature. Subsequently, a standard solution of ABTS was prepared with MeOH to an absorbance of 1.1 ± 0.02 at a wavelength of 734 nm using a BioTek Epoch 2 plate reader (BIOTEK, Winooski, VT, USA). The anti-radical reaction was measured for 1 h in the dark at room temperature. To the resulting solution, 270 µL of adjusted ABTS and 30 µL of the *P. hysterophorus* L. EOs, at different concentrations (8000, 6000, 4000, 2000 µg/mL), were added. Trolox was used as a positive control and MeOH as a blank. The results are expressed in terms of 50% radical scavenging concentration (IC_50_).

### 4.6. Statistical Analysis

All the procedures were performed in triplicate. The principal component analysis (PCA) was performed to analyze the variability in the chemical composition of EOs across the three collection sites. To analyze the effects of locality on chemical composition, a Permutational Multivariate Analysis of Variance (PERMANOVA) was performed using Bray–Curtis distances and 9999 Monte Carlo permutations. One-way ANOVA was used to test whether the AChE-inhibitory activity of *P. hysterophorus* L. differed significantly between the populations. The underlying assumptions of normality were tested using the Shapiro test (*p*-value < 0.05). On the other hand, a non-parametric Kruskal–Wallis test was carried out to test whether the antioxidant activity of EOs of *P. hysterophorus* L. evaluated by ABTS and DPPH methods varied significantly among the three populations. The PERMANOVA analysis was conducted with the ‘vegan’ package [43]. All the analyses were conducted utilizing the statistical software R 3.2.2. [44].

## 5. Conclusions

The present study provides a detailed analysis of the chemical composition and biological activity of EOs extracted from *Parthenium hysterophorus* L., with germacrene D, myrcene, (*E*)-β-ocimene, (*E*)-caryophyllene and (*E*, *E*)-α-farnesene identified as the most abundant compounds. The EOs showed moderate antioxidant activity, with IC_50_ values ranging from 14.50 to 158.43 µg/mL. All EOs showed inhibitory activity against AChE enzymes, with the QUI sample showing the highest potency (IC_50_ = 10.69 µg/mL). Multivariate statistical analysis including PCA and PERMANOVA revealed variations in chemical composition among the three collection sites. These differences are likely influenced by environmental factors such as altitude, temperature and rainfall. These findings support the potential use of *P. hysterophorus* EOs as possible complementary agents in managing neurodegenerative diseases such as Alzheimer’s. However, further mechanistic and in vivo studies are needed, as is further research, to isolate and characterize bioactive compounds and explore their therapeutic applications. 

## Figures and Tables

**Table 1:** Qualitative (GC-MS) and quantitative (GC-FID) chemical composition of *P. hysterophorus* EO on 5-phenyl-methylpolysiloxane column.

				5-Phenyl-methylpolysiloxane		
N^◦^	Compounds	LRI ^a^	LRI ^b^	ESP	LOJ	QUI
				% ± SD		
1	tricyclene	921	923	0.07 ± 0.04	n.d.	0.05 ± 0.02
2	α-pinene	932	933	0.51 ± 0.05	0.29 ± 0.06	0.38 ± 0.06
3	camphene	946	950	1.18 ± 0.07	0.52 ± 0.08	n.d.
4	α-fenchene	945	951	n.d.	n.d.	0.68 ± 0.08
5	sabinene	969	974	0.30 ± 0.05	0.14 ± 0.04	0.14 ± 0.04
6	β-pinene	974	980	1.50 ± 0.08	0.68 ± 0.10	0.71 ± 0.08
7	myrcene	988	992	11.33 ± 1.06	13.95 ± 13.15	13.36 ± 0.26
8	limonene	1024	1032	n.d.	0.20 ± 0.05	0.22 ± 0.06
9	β-phellandrene	1025	1034	0.43 ± 0.07	0.26 ± 0.06	0.22 ± 0.06
10	(*Z*)-β-ocimene	1032	1039	0.10 ± 0.04	0.08 ± 0.04	0.09 ± 0.04
11	(*E*)-β-ocimene	1044	1049	10.21 ± 0.10	10.51 ± 10.11	8.54 ± 0.14
12	α-fenchocamphorone	1104	1116	0.30 ± 0.06	0.22 ± 0.04	0.28 ± 0.06
13	bornyl acetate	1284	1292	1.25 ± 0.05	1.25 ± 1.10	0.63 ± 0.10
14	δ-elemene	1335	1337	n.d.	0.23 ± 0.05	0.29 ± 0.06
15	silphinene	1345	1350	n.d.	n.d.	0.06 ± 0.02
16	cyclosativene	1369	1372	0.39 ± 0.06	0.18 ± 0.03	0.11 ± 0.03
17	α-ylangene	1373	1378	0.45 ± 0.09	n.d.	n.d.
18	α-copaene	1374	1381	n.d.	0.20 ± 0.04	0.22 ± 0.04
19	daucene	1380	1380	n.d.	0.10 ± 0.03	n.d.
20	β-bourbonene	1387	1386	0.11 ± 0.04	0.11 ± 0.03	n.d.
21	β-cubebene	1387	1390	0.61 ± 0.04	0.44 ± 0.07	n.d.
22	sibirene	1400	1391	n.d.	n.d.	0.44 ± 0.08
23	β-elemene	1389	1392	n.d.	0.14 ± 0.04	0.11 ± 0.03
24	sativene	1390	1395	0.03 ± 0.03	n.d.	n.d.
25	β-longipinene	1400	1399	0.04 ± 0.04	n.d.	n.d.
26	α-cis-bergamotene	1411	1416	n.d.	n.d.	0.04 ± 0.03
27	(*E*)-caryophyllene	1417	1424	7.93 ± 0.08	3.98 ± 3.13	5.37 ± 0.12
28	α-trans-bergamotene	1432	1431	0.26 ± 0.07	0.20 ± 0.03	0.04 ± 0.03
29	γ-elemene	1434	1435	n.d.	n.d.	0.27 ± 0.07
30	α-guaiene	1437	1439	n.d.	0.08 ± 0.03	0.05 ± 0.03
31	6.9-guaiadiene	1442	1445	0.13 ± 0.04	n.d.	0.19 ± 0.05
32	(*E*)-β-farnesene	1454	1455	0.27 ± 0.07	0.33 ± 0.06	0.56 ± 0.10
33	α-humulene	1452	1461	1.40 ± 0.12	0.84 ± 0.09	0.08 ± 0.12
34	9-epi-(*E*)-caryophyllene	1464	1465	0.05 ± 0.03	n.d.	n.d.
35	dauca-5,8-diene	1471	1478	n.d.	0.10 ± 0.04	0.19 ± 0.05
36	γ-muurolene	1480	1481	0.22 ± 0.07	0.14 ± 0.04	0.15 ± 0.04
37	germacrene D	1493	1489	35.27 ± 1.41	28.30 ± 28.30	32.22 ± 0.20
38	bicyclogermacrene	1500	1502	1.89 ± 0.05	1.42 ± 1.12	1.57 ± 0.12
39	(*E*, *E*)-α-farnesene	1505	1507	4.99 ± 0.08	3.64 ± 3.14	6.77 ± 0.16
40	δ-amorphene	1511	1509	n.d.	2.82 ± 2.12	3.07 ± 0.16
41	α-bulnesene	1509	1514	0.22 ± 0.06	n.d.	0.07 ± 0.04
42	γ-cadinene	1513	1520	n.d.	n.d.	0.48 ± 0.12
43	δ-cadinene	1522	1524	1.35 ± 0.05	0.87 ± 0.12	0.55 ± 0.10
44	β-sesquiphellandrene	1521	1529	0.41 ± 0.06	0.29 ± 0.07	n.d.
45	γ-cuprenene	1532	1539	0.21 ± 0.06	0.06 ± 0.02	n.d.
46	α-copaen-11-ol	1539	1539	n.d.	n.d.	0.04 ± 0.03
47	trans-cadinene ether	1557	1555	n.d.	n.d.	1.19 ± 0.12
48	(*E*)-nerolidol	1561	1567	0.70 ± 0.05	0.96 ± 0.11	0.15 ± 0.05
49	1α,10α-epoxy-amorph-4-ene	1570	1575	n.d.	0.53 ± 0.09	n.d.
50	spathulenol	1577	1576	0.35 ± 0.07	n.d.	n.d.
51	not identified	1582		n.d.	1.95 ± 1.10	n.d.
52	β-copaen-4-α-ol	1590	1582	1.27 ± 0.06	n.d.	1.02 ± 0.14
53	not identified	1590		1.16 ± 0.04	1.59 ± 1.14	1.79 ± 0.14
54	cis-dihydro-mayurone	1595	1594	1.51 ± 0.07	0.61 ± 0.11	n.d.
55	isolongifolan-7-α-ol	1618	1614	2.05 ± 0.05	8.26 ± 8.12	2.33 ± 0.16
56	β-atlantol	1608	1618	n.d.	n.d.	0.85 ± 0.12
57	1,10-di-epi-cubenol	1618	1623	n.d.	0.54 ± 0.11	0.15 ± 0.05
58	epi-α-cadinol	1638	1634	0.21 ± 0.07	0.45 ± 0.11	0.27 ± 0.08
59	muurola-4,10(14)-dien-1-β-ol	1630	1639	n.d.	n.d.	0.02 ± 0.02
60	β-acorenol	1636	1639	0.26 ± 0.06	n.d.	n.d.
61	cis-guaia-3,9-dien-11-ol	1648	1654	0.87 ± 0.06	0.90 ± 0.12	n.d.
62	14-hydroxy-9-epi-(*E*)-caryophyllene	1668	1663	0.05 ± 0.04	0.37 ± 0.09	0.03 ± 0.02
63	germacra-4(15),5,10(14)-trein-1- α-ol	1685	1681	5.18 ± 0.08	8.00 ± 7.15	5.69 ± 0.09
64	junicedranol	1692	1689	n.d.	0.57 ± 0.11	0.41 ± 0.06
65	eudesma-4(15),7-dien-1β-ol	1687	1698	0.64 ± 0.01	0.82 ± 0.08	0.64 ± 0.09
66	14-hydroxy-α-humulene	1713	1709	n.d.	n.d.	0.09 ± 0.04
67	14-hydroxy-α-muurolene	1779	1781	n.d.	0.11 ± 0.05	n.d.
68	(*E*)-isovalencenol	1793	1786	n.d.	n.d.	0.05 ± 0.03
69	14-hydroxy-δ-Cadinene	1803	1797	n.d.	n.d.	0.04 ± 0.03
70	n-heneicosane	2100	2095	n.d.	0.04 ± 0.02	n.d.
Total identified (%)				96.51	94.73	94.06
Alcohols (%)				3.87	11.04	4.07
Ketones (%)				1.51	0.61	0
Monoterpene hydrocarbons (%)				25.64	26.63	24.37
Sesquiterpene hydrocarbons (%)				56.23	44.47	54.81
Oxygenated monoterpenoids (%)				1.55	1.47	0.91
Oxygenated sesquiterpenes (%)				7.71	10.517	9.9

LRI ^a^ = linear retention index determined; LRI ^b^ = linear retention index according to Adams [20]; %± SD = percentage and standard deviation. Values are the mean of three determinations. n.d. = not detectable.

**Table 2:** Acetylcholinesterase-inhibitory and antioxidant activity of *Parthenium hysterophorus* essential oils from Espíndola (ESP), Loja (LOJ) and Quilanga (QUI).

Sample	AChE IC_50_ (µg/mL)	DPPH IC_50_ (µg/mL)	ABTS IC_50_ (µg/mL)
ESP	14.78 ± 1.02	93.16 ± 1.10	16.38 ± 0.7
LOJ	16.65 ± 1.02	158.43 ± 1.61	36.18 ± 1.15
QUI	10.69 ± 1.02	92.98 ± 1.06	14.50 ± 0.7
Donepezil (AChE control)	12.40 ± 1.35	—	—
Trolox (antioxidant control)	—	35.54 ± 1.04	29.09 ± 1.05

The values represent the mean ± SD, *n* = 3

## Data Availability

The datasets presented in this article are not readily available because they are part of an ongoing study. Requests to access the datasets should be directed to the corresponding author.

## Supplementary Materials

The following supporting information can be downloaded at https://www.mdpi.com/article/10.3390/molecules30132712/s1, Figure S1: Myrcene mass spectrum; Figure S2: (*E*)-β-ocimene mass spectrum; Figure S3: (*E*)-caryophyllene mass spectrum; Figure S4: Germacrene D mass spectrum; Figure S5: (*E*, *E*)-α-farnesene mass spectrum.
