# Supplementary material for: Chemical Composition, Acetylcholinesterase-Inhibitory Potential and Antioxidant Activity of Essential Oils from Three Populations of Parthenium hysterophorus L. in Ecuador
Source: Molecules. 2025 Jun 24;30(13):2712. doi: 10.3390/molecules30132712 (PMC12250999; doi:10.3390/molecules30132712)

Supplementary Material

Figure S1. Myrcene mass spectrum

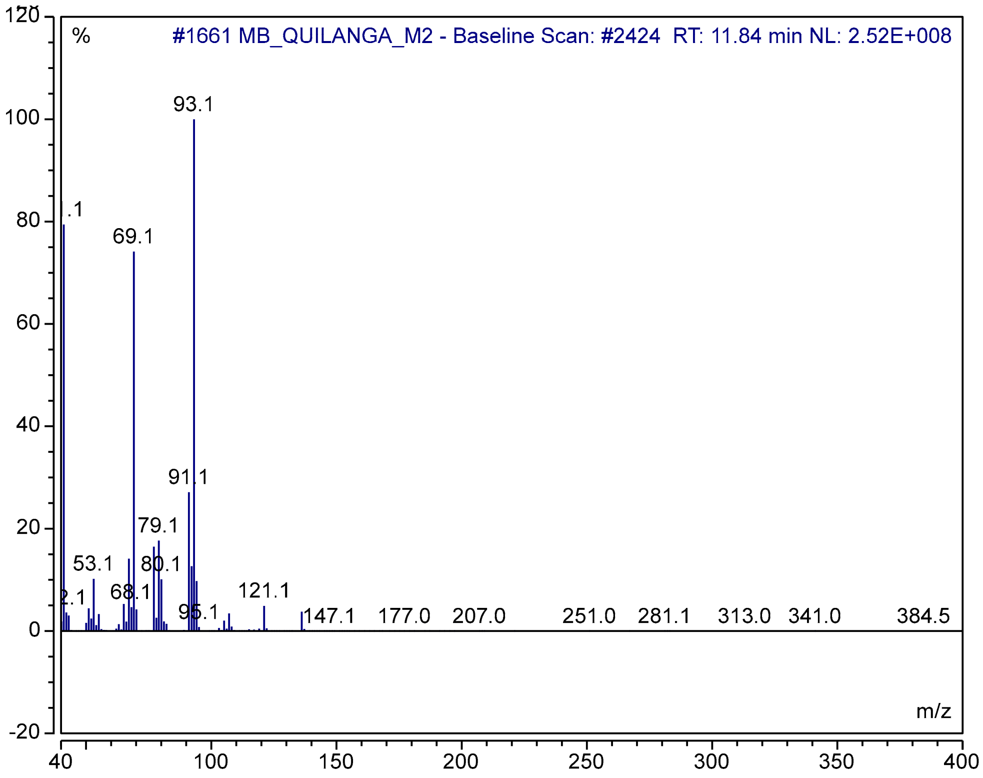

Figure S2. (E)-β-ocimene mass spectrum

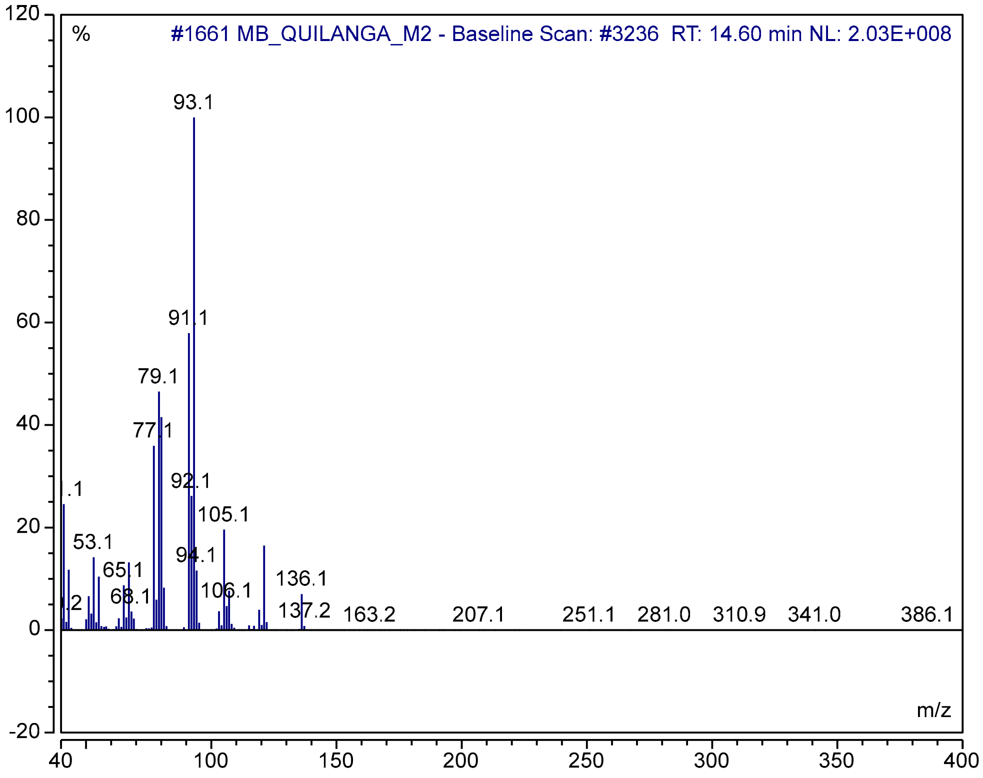

Figure S3. (E)-caryophyllene mass spectrum

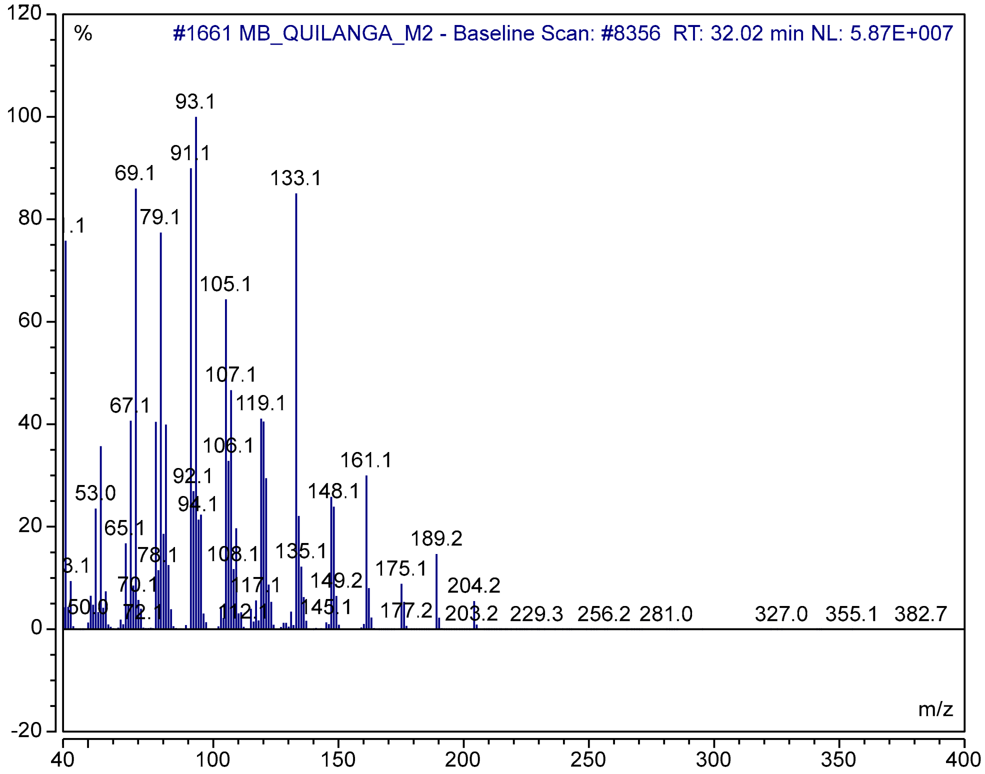

Figure S4. Germacrene D mass spectrum

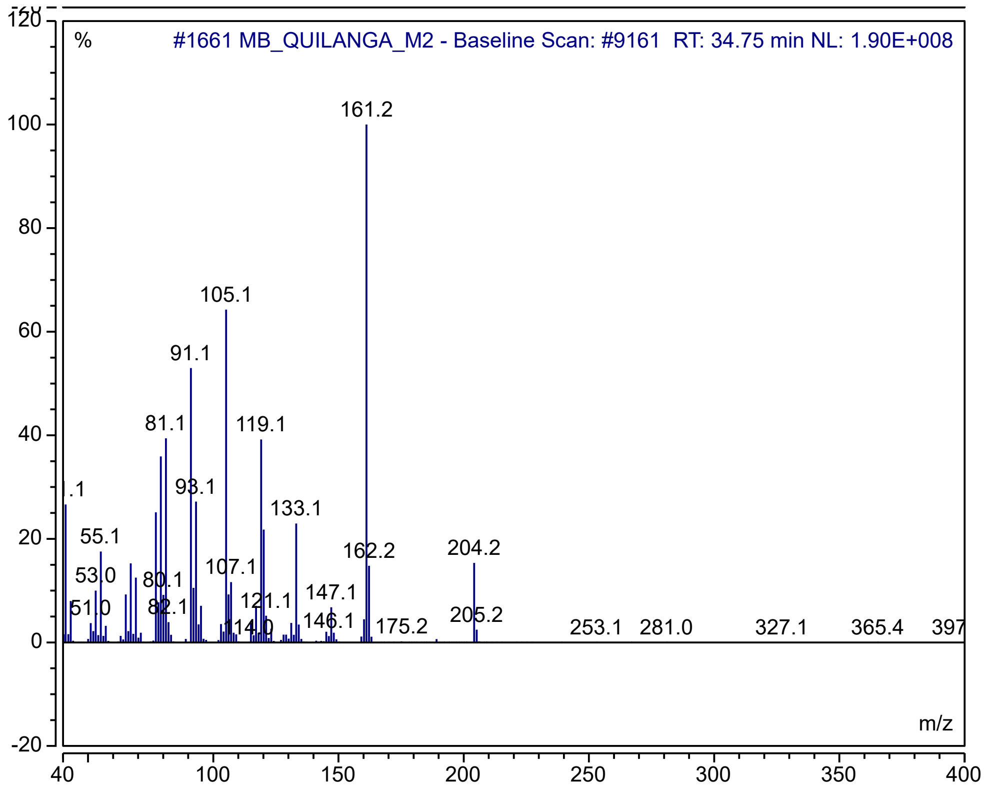

Figure S5. (E, E)-α-farnesene mass spectrum

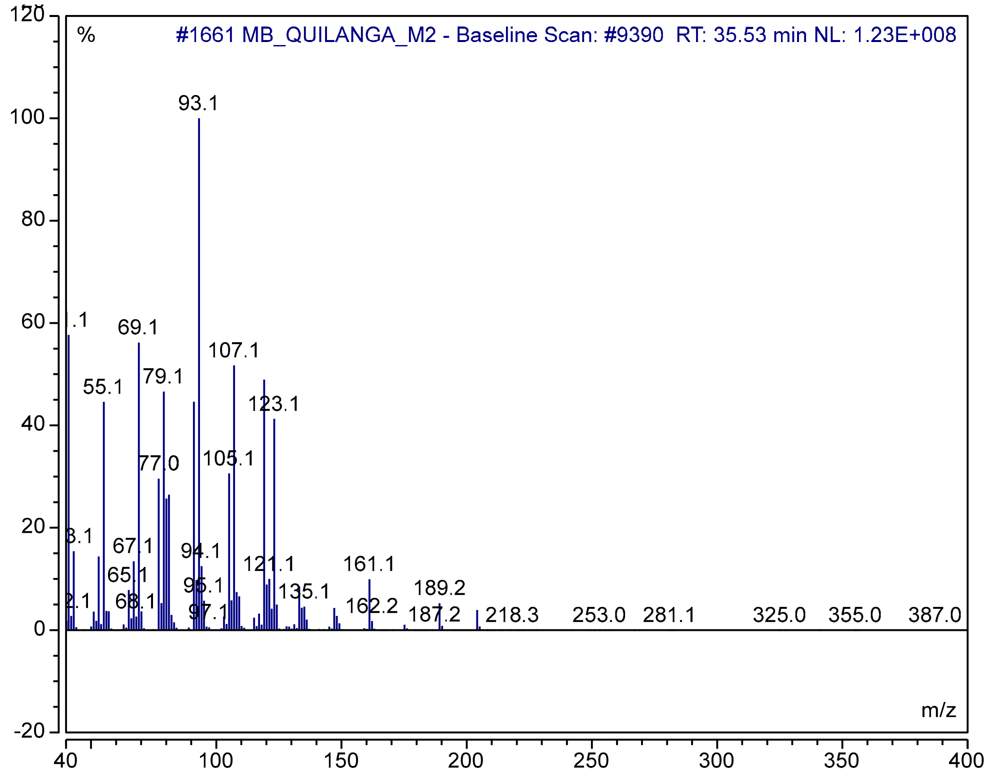

Supplement: Supplementary file 1 [file molecules-30-02712-s001.zip › molecules-3638085-supplementary.pdf]
